# Editorial: The Separation and Removal of Inorganic Ions and Organics From Aqueous Solutions

**DOI:** 10.3389/fchem.2021.757447

**Published:** 2021-11-01

**Authors:** Shenxu Bao, Hong Peng, Feng Rao, Wencai Zhang

**Affiliations:** ^1^ School of Resources and Environmental Engineering, Wuhan University of Technology, Wuhan, China; ^2^ School of Chemical Engineering, The University of Queensland, Brisbane, QLD, Australia; ^3^ Institute Investigation Metallurgy and Materials, Universidad Michoacana de San Nicolas de Hidalgo, Morelia, Mexico; ^4^ Department of Mining and Minerals Engineering, Virginia Polytechnic Institute and State University, Blacksburg, VA, United States

**Keywords:** editorial, separation and recovery, extraction, removal, capacitive deionization, hydrometallurgy

**Editorial on the Research Topic**

**The Separation and Removal of Inorganic Ions and Organics From Aqueous Solutions**

Dear Colleagues,

This issue of *The Separation and Removal of Inorganic Ions and Organics from Aqueous Solutions* contains nine papers from the regular submissions to the Frontiers in Chemistry journal.

*The separation and removal of inorganic ions and organics from aqueous solutions* is an essential question in many fields. With the development of modern science and engineering, there are various techniques that have been developed and applied in the separation and removal of ions and organic pollutants from aqueous solutions. These techniques aim at solving separation problems encountered in the emerging technologies including fields such as any separation and/or purification of liquids, green technology, and resource recovery and recycling. Of particular interest is that the separation and removal of wanted metals from leaching solutions, which is an indispensable procedure for most hydrometallurgy processes. Ion exchange and solvent extraction, the well-established methods for resource extraction from aqueous solutions, have become effective and versatile techniques to recover wanted metals from low-grade ore or secondary resources.

In this Research Topic, Laxman et al. used the activated carbon cloth (ACC) electrode-based capacitive deionization (CDI) device to remove ionic contaminants in water, and they studied the effect of ion concentrations on the electrosorption and disinfection functions of the CDI device. This technology has a minimal impact on the environment and can be recommended as a green alternative for water treatment for deionization and microbial disinfection. The electrochemical treatment of wastewater is widely used for cleaning due to its efficiency. A comparative study on the electrochemical treatment of cyanide wastewater by two-dimensional (2D) and three-dimensional (3D) electrochemical systems was carried out (Lei and Song). The 3D electrode electrochemical system treatment technique has a bright prospect for the removal of organic material and heavy metal ions from industrial wastewater of chemical, metallurgical, and material industries. Li et al. synthesized 1-(2-hydroxyphenyl) dec-2-en-1-one oxime (HPDO) using 2-hydroxy acetophenone and octanal. The research results indicated that HPDO is a special collector for malachite flotation, whose collecting ability was improved as desired. In addition, they reported in 2021 that tert-octylsalicylaldoxime with its new structure exhibited excellent extraction ability and selectivity for Cu(II), and can be successfully used to recover copper from copper-nickel alloy electroplating wastewater. This product has the potential to be used as a powerful copper extractant in future. To overcome the problems of arsenic separation and enrichment from an alkaline leaching solution in arsenic-containing dust, a CO32--type tri-n-octylmethyl-ammonium chloride (TOMAC) was proposed to extract thioarsenite. This study provides an alternative for the removal of arsenic via alkaline leaching from high arsenic flue dust produced by heavy metal smelting (Yan et al.). The separation of Cr^3+^/Fe^3+^ from the acid leaching solution of electroplating sludge by the selective complex precipitation has been successfully achieved and the developed procedure will help to promote further research in this area (Jinhui et al.). The selective leaching of tellurium from telluride-type gold concentrate by the Na_2_S + NaOH cooperative leaching process provides new ideas for the separation and extraction of tellurium, a rare element, from telluride-type gold concentrate (Yang et al.). Zhuo et al. reported the leaching process of weathered crust elution-deposited rare Earth ore with formate salts. Formate salts are a green and sustainable rare Earth leaching agent, which can strengthen the rare Earth leaching process and weaken the hydration of clay minerals for preventing landslides. The ion-exchange mechanism in the leaching process of weathered crust elution-deposited rare Earth ores with different leaching agents was studied, which can improve the rare Earth leaching efficiency and mine safety (Zhang et al.).

With this Research Topic, we have collated new insights about the separation and removal of inorganic ions and organics from aqueous solutions. Studies addressed the application of CDI technology and electrochemicals in water treatment, the leaching process of telluride-type gold concentrate and weathered crust elution-deposited rare Earth ore, and the extraction of Cu(II) and thioarsenite with new extractants, etc. The aim of this Research Topic is to provide an interdisciplinary platform for researchers to exchange and share their experiences and latest achievements in various aspects of the separation and purification field and, in this way, promote the progress in theory and technology. The published papers present new ideas, research, and technologies, which can lead to not only economic but more environmentally friendly processes.

